# Model systems for studying cell adhesion and biomimetic actin networks

**DOI:** 10.3762/bjnano.5.131

**Published:** 2014-08-01

**Authors:** Dorothea Brüggemann, Johannes P Frohnmayer, Joachim P Spatz

**Affiliations:** 1 Department of New Materials and Biosystems, Max Planck Institute for Intelligent Systems, Heisenbergstr. 3, D-70569 Stuttgart, Germany; 2 Department of Biophysical Chemistry, University of Heidelberg, INF 253, D-69120 Heidelberg, Germany

**Keywords:** actin network, cell adhesion, giant unilamellar vesicle, integrin, lipid bilayer, synthetic cell, protein reconstitution, talin

## Abstract

Many cellular processes, such as migration, proliferation, wound healing and tumor progression are based on cell adhesion. Amongst different cell adhesion molecules, the integrin receptors play a very significant role. Over the past decades the function and signalling of various such integrins have been studied by incorporating the proteins into lipid membranes. These proteolipid structures lay the foundation for the development of artificial cells, which are able to adhere to substrates. To build biomimetic models for studying cell shape and spreading, actin networks can be incorporated into lipid vesicles, too. We here review the mechanisms of integrin-mediated cell adhesion and recent advances in the field of minimal cells towards synthetic adhesion. We focus on reconstituting integrins into lipid structures for mimicking cell adhesion and on the incorporation of actin networks and talin into model cells.

## Review

### Introduction

Since Hooke first described a biological cell in 1665 tremendous progress has been made in understanding the basic functions of living cells including signalling pathways, gene regulation and the molecular structure of cellular components. However, with each new discovery it becomes clearer that cells are very complex, active systems with many parts interlinked to each other. This complexity makes it very difficult to selectively study a single aspect of natural cells. In recent years, minimal cells with reduced molecular complexity have gained increasing importance as model systems for living cells. Such synthetic cells often consist of lipid vesicles with various incorporated proteins, which are used to study biochemical reactions and self-assembly processes in a controlled environment with reduced molecular complexity [1–2]. We here review previous advances in the development of model cells with reconstituted integrin and incorporated actin networks. We also report on the fundamental mechanisms of integrin-mediated cell adhesion and the interaction of talin with lipid vesicles.

### 1. The role of integrins in cell adhesion

**Figure 1:**
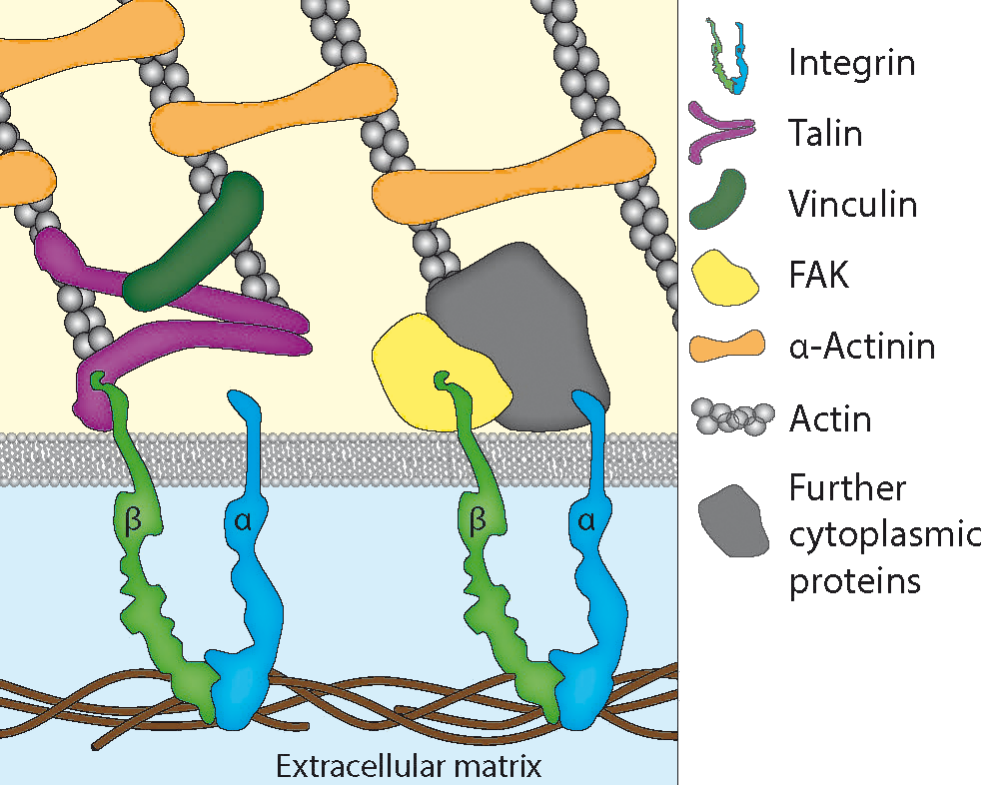
Schematic view of active integrin molecules linking the ECM to the actin cytoskeleton. The heads of the integrin molecules attach directly to their ligand molecules in the ECM; the intracellular tail of integrin binds to proteins like talin and FAK. On the intracellular side, active talin dimers also bind to filamentous actin. Other proteins like FAK form an indirect linkage to the actin cortex together with further cytoplasmic proteins. These intracellular anchor proteins, which include vinculin and α-actinin, help to regulate and reinforce the actin–integrin linkage.

Cellular adhesion is an important mechanism, which enables cells to bind to the extracellular matrix and to surrounding cells. This process is crucial in regulating cell shape and for maintaining cell viability, migration, and tissue integrity [3]. Cell–cell and cell–substrate adhesion are mediated by different proteins, the cell adhesion molecules (CAMs). A very important group of CAMs is the integrin family, which functions both as cell–substrate and cell–cell adhesion receptors [4]. All connective tissues are supported by an extracellular protein structure, the extracellular matrix (ECM). For the first time, in 1986 an integrin was reported to link the intracellular cytoskeleton with the ECM. The name “integrins” was given to these receptors to denote their integral membrane nature and importance for the integrity of the cytoskeleton–ECM linkage [5]. Integrins are heterodimeric transmembrane glycoproteins consisting of noncovalently associated α and β subunits. In humans 24 different αβ permutations of such heterodimers exist, each of which can bind to a wide variety of specific ligands in the ECM [6–8]. Integrins contain short cytoplasmic tails and large extracellular domains, which enable the bi-directional transmission of forces across the plasma membrane [9]. Besides their anchorage function, integrins also provide spatial information on the environment and the adhesive state of the cell by transmitting chemical signals into the cytoplasm [10–11]. In addition to this outside-in signalling process integrins can undergo conformational changes, which are called inside-out activation. These changes are primarily induced by talin, a major actin-binding protein, which binds to the cytoplasmic β tails of integrins, thus activating the molecule (Figure 1). When integrin is present in its activated state it shows a higher affinity for ligands on the extracellular side of the cell membrane [12–15]. Many of these protein ligands in the ECM, for instance fibronectin, vitronectin, fibrinogen or laminin, contain the tripeptide sequence arginine-glycine-aspartic acid (RGD), which is specifically recognised by most integrins [3,7–8]. The affinity as well as the specificity of integrins to bind to their ligands in the ECM can also be increased by the presence of divalent ions such as manganese or calcium [16].

Cellular adhesion strength is mostly controlled by the intermolecular spacing of the adhesion receptors rather than by their density [17]. This result was obtained from different studies using highly ordered gold nanoparticles, which were functionalised with RGD peptides. The adhesive gold nanoparticles had a diameter below 8 nm, which allowed the binding of one integrin molecule per RGD-functionalised nanoparticle [18]. If the distance between adjacent integrin-binding gold nanoparticles was less than 70 nm, cell adhesion was found to be reinforced [17]. With nanoparticle spacings above 90 nm, focal contact formation and cell spreading were inhibited [19]. For a large variety of cells a universal length scale of 58 to 73 nm was found, which provided integrin clustering and activation, hence leading to effective cell adhesion [18]. These results showed that integrin signalling enables cells to amplify small environmental differences in adhesive cues to large differences in adhesion strength.

As the cortical cytoskeleton of all cells is formed by the assembly of actin microfilaments, their linkage to adhesion-mediating proteins like integrin is important for cell shape and migration. The actin-based motility of cells is driven by myosin, a molecular motor, which converts chemical energy in the form of ATP to mechanical energy, thus generating force and movement [20]. When integrins have created a cell–ECM contact they cluster and recruit the cytoplasmic protein focal adhesion kinase (FAK), which generates stable anchoring cell–matrix junctions, the focal adhesions. These adhesion sites are crucial for the cytoskeleton, environmental sensing and cellular motility [6,21–22]. Besides talin, several other proteins including vinculin and α-actinin are well known to form integrin–actin linkages [23]. To date, the signalling network of potential integrin–actin linkages contains 156 components and 690 interactions, which have been summarised in a functional atlas of the integrin adhesome (http://www.adhesome.org) by Geiger and co-workers [24–25].

Further fundamental roles of integrin molecules in signalling and other cellular functions have been studied by using various knockdown animal models [7,26–28]. Studies on the molecular structure of integrins over the past 28 years have revealed that these adhesion molecules do not only have a vital function in cell health. They also contribute to the progression of many diseases, in which cell adhesion and migration are impaired due to alterations in the expression of integrins and their functionality. To date, integrins have already been identified as key factors in inflammation, thrombosis, cancer, fibrosis, autoimmune disorders, and infectious diseases [29–31]. These discoveries have brought integrins into the focus of pharmacological research for the development of anti-integrin drugs. At least three different integrins have been identified as therapeutic targets, and around 260 anti-integrin drugs have already entered clinical trials to date [30].

Lipid vesicles with incorporated integrin are a valuable model system to study the complex processes involved in cell adhesion with reduced molecular complexity. Talin is important for the activation of integrin and also creates a stable linkage between integrin and the cytoskeletal protein actin. Hence, these three proteins are important candidates to be incorporated into minimal cells, which mimic cell adhesion and shape.

### 2. Integrin reconstitution into lipid membranes

Membrane proteins and their specific impact on cell adhesion can be difficult to study in their natural environment due to the complexity of native cell membranes and interfering interactions with other membrane components and the cellular environment [32]. Over the past years, research on cell adhesion mechanisms has strongly benefited from the possibility to isolate membrane proteins, such as integrin, from cells and to embed them into lipid structures ranging from planar bilayers and small liposomes to giant unilamellar vesicles. This procedure has enabled studies on the functional properties of membrane proteins in a defined environment with reduced molecular complexity. We here review recent advances on integrin reconstitution in lipid structures and the significance of these model systems for understanding integrin-mediated cell adhesion.

#### 2.1 Integrin incorporation into small lipid vesicles and planar bilayers

The successful reconstitution of functional integrins into small phospholipid vesicles was first achieved in 1985 by Parise et al. They worked with integrin α_IIb_β_3_, which they purified from blood platelets [33–34]. This transmembrane glycoprotein is a major receptor of the platelet plasma membrane, which is required for platelet aggregation and has been well characterised. The liposomes, into which the integrins were incorporated, had diameters of 40 ± 8 nm and can be characterised as small liposomes. Integrin reconstitution was carried out by a detergent-dialysis method. During reconstitution the purified integrins were solubilized in octyl glucoside and added to different nitrogen dried phospholipids. Afterwards, the protein–lipid mixture was dialysed against a 1000 fold excess of the integrin buffer without octyl glucoside [34]. This procedure yielded proteoliposomes with functional integrin for the first time, as it was confirmed by specific binding to fibrinogen [33].

**Figure 2:**
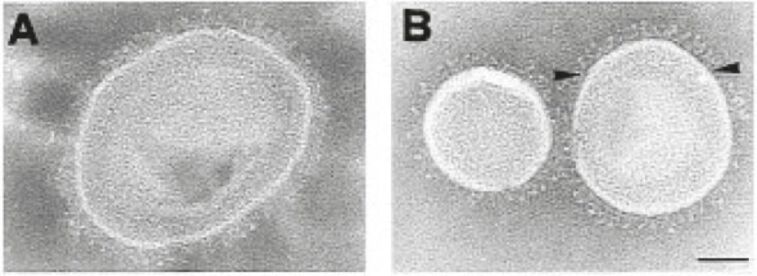
Cryoelectron micrographs of negatively stained DMPG/DMPC vesicles containing integrin α_IIb_β_3_. Negatively stained vesicles with (A) high and (B) low surface density of integrin α_IIb_β_3_. The scale bar represents 100 nm. (Reprinted with permission from [40]. Copyright (1997) American Chemical Society.)

Triton X-100 is another detergent that is widely used for the reconstitution of numerous membrane proteins into liposomes since the 1980s [32,35–36]. This detergent has the tendency to form large micelles, which cannot be removed by dialysis. An efficient way of removing Triton X-100 is to use non-polar Bio-Beads, which are macroporous divinylbenzene cross-linked polystyrene beads. Detergents adsorb to these porous beads by hydrophobic bonds and can be removed from the protein solution in this way [37–38]. Müller and co-workers were the first to use this detergent removal method for reconstituting integrin α_IIb_β_3_ from blood platelets and α_1_β_1_ from chicken gizzard into lipid vesicles. Successful integrin incorporation into liposomes of 100 to 200 nm in diameter was confirmed by negative staining in cryoelectron microscopy (Figure 2). Proteoliposomes with integrin α_IIb_β_3_ were also used to form planar lipid bilayers by vesicle fusion. With these planar integrin–lipid bilayers Müller et al. provided evidence that the binding of integrin α_IIb_β_3_ to fibrinogen is a biphasic process consisting of a reversible first and a second irreversible step [39].

Erb and Engel later showed by cryoelectron and fluorescence microscopy that activated integrin α_IIb_β_3_ reconstituted into liposomes and planar bilayers was present in a nonclustered state and was equally distributed within the membrane. When fibrinogen was bound to the proteolipid structures the integrins became immobile and transformed into clusters, thus passing into the previously observed irreversibly bound state [40]. Detailed protocols for the reconstitution of functional integrin into liposomes and planar bilayers of Erb and Engel have been summarised later together with several methods to analyse proteoliposome formation [41]. Recently, the activation of integrin α_IIb_β_3_ reconstituted into liposomes was also studied by cryoelectron tomography [42]. With this technique it was possible to show that integrin activation with Mn^2+^ does not result in a height change of the integrin molecule. This observation was not as predicted by the switchblade model and is more consistent with the deadbolt model.

Goennenwein et al. reconstituted integrin α_IIb_β_3_ into supported lipid bilayers to measure their adhesion force against RGD-peptide carrying giant vesicles. With this setup a simple and powerful tool was found to quantify the binding energy of different integrin–ligand pairs under bioanalogue conditions [43]. This system was developed further to facilitate the mobility of the integrin receptors within the supported lipid bilayer. Thus, it was shown that integrin mobility controls the force-induced growth of cell adhesion domains, which play an important role in mechanosensing of living cells [44].

In a study by Sinner et al. the integrins α_V_β_3_ and α_1_β_1_ were incorporated into planar lipid membranes, which were obtained by vesicle spreading. With surface plasmon-enhanced fluorescence spectroscopy and surface plasmon spectroscopy the structural and functional integrity of the embedded receptors could be confirmed over a time period of three days [45]. In binding experiments with various ECM ligands hardly any nonspecific binding was observed. Furthermore, Sinner and co-workers succeeded in regenerating the binding capacity of the artificial membranes by dissociating the integrin–ECM ligand complexes with ethylenediaminetetraacetate (EDTA). They concluded that integrin-functionalised membranes are well qualified as sensing devices for the detection of sensible ligand–receptor interactions.

#### 2.2 Giant unilamellar vesicles with reconstituted membrane proteins

A model system that is much closer to natural cells than planar lipid membranes and small liposomes are giant unilamellar vesicles (GUVs). GUVs have diameters of 1 to 100 μm and enclose an aqueous medium. Their shell consists of only one lipid bilayer, like the membrane of natural cells. With these attributes GUVs have gained increasing importance as bottom-up model systems in synthetic biology over the past years. GUVs can be used to study cellular functions and the interplay between various proteins, which are incorporated in the lipid structures [1–2]. Other attempts in synthetic biology aim at building artificial cell systems from polymersomes. Although protocells prepared from polymer membranes have a higher stability than lipid vesicles they lack biorelevance. Furthermore, polymer membranes are much thicker than lipid bilayers, which are only 4 nm thick [46]. For the preparation of GUVs several methods exist, amongst which are electroformation on platinum wires or indium tin oxide (ITO) electrodes as well as spontaneous swelling. The specifics of GUVs, their detailed preparation methods and wide ranging applications are reviewed by P. Walde et al. [47].

Several membrane proteins have already been incorporated into GUVs over the past years. Girard and co-workers first reconstituted Ca^2+^-ATPase from the sarcoplasmic reticulum and the H^+^ pump bacteriorhodopsin into liposomes of 0.1 to 0.2 μm in size [48]. The liposomes were partially dried; the subsequent electroformation process on ITO yielded protein-GUVs with diameters between 5 and 100 μm. Both membrane proteins were homogeneously incorporated in the membranes and biologically active as demonstrated by Ca^2+^ or H^+^ pumping. Functionally active aquaporins have also been embedded into GUV membranes either by mixing protein-reconstituted liposomes of 0.1 to 0.2 μm diameter with a lipid-containing oil phase (lipid cosolvent method) [49] or by swelling of a tissue-like giant vesicle film [50]. For the first time, Dezi et al. recently incorporated different transmembrane proteins like bacteriorhodopsin and the ferrichrome transport protein FhuA into GUVs with lipid mixtures representative of cellular plasma membranes [51]. Reconstitution was either performed with proteins solubilised in detergent micelles, with proteoliposomes or purified native membranes. This method proved to be highly versatile and can in future be applied to a large range of other transmembrane proteins, such as integrins.

**Figure 3:**
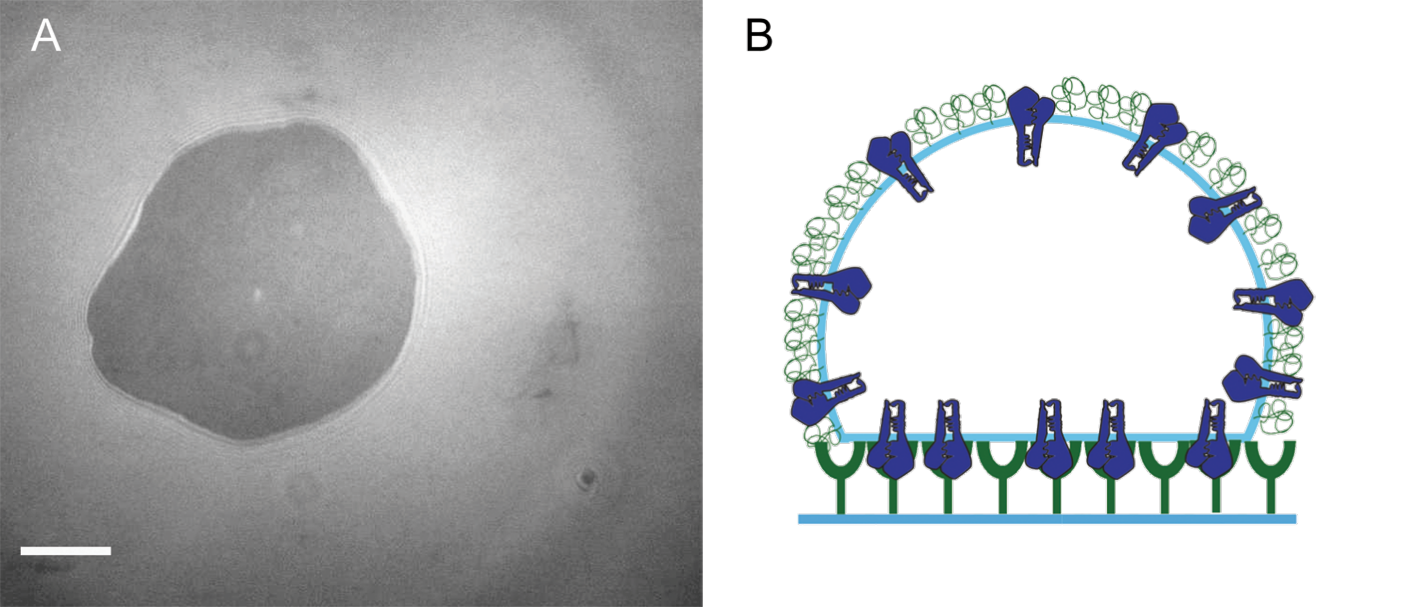
GUVs containing integrins interacting with a fibrinogen-coated substrate: (A) adhesion is detected with RICM (black patch), (B) Scheme of the minimal cell system. The scale bar represents 10 μm (Reprinted with permission from [54]. Copyright (2009) Elsevier Ltd.)

Despite the large variety of complex membrane proteins, which have already been incorporated into GUVs, such as functional ion channels [52–53], the only existing work of integrin reconstitution in GUVs to date was published in 2008. Streicher et al. developed a novel biomimetic system based on giant vesicles that mimicked the first steps of integrin-mediated cell adhesion [54]. GUVs were produced from small liposomes by electroformation and had a diameter of 20 to 40 μm. The successful incorporation of partly fluorescently labelled integrin α_IIb_β_3_ into the GUVs was confirmed by confocal microscopy. Binding experiments of integrin-GUVs on surfaces and quantum dots coated with RGD ligands revealed that the incorporated integrins were biologically active. In reflection interference contrast microscopy (RICM) experiments (Figure 3) it was observed that integrin-GUVs adhered to fibrinogen surfaces in a two-step spreading process without any enrichment of integrins in the adhesive patches. From these observations Streicher et al. concluded that the role of the actin cytoskeleton in natural cells is to stabilise more integrins in the adhesion zones to form focal adhesion spots by recruiting FAK and other cytoplasmic proteins.

### 3. Biomimetic actin cortices in lipid vesicles

Due to the importance of actin in cell adhesion several studies have already been presented on the incorporation of this protein into liposomes and GUVs to build a biomimetic system that mimics cell adhesion, the formation of a cytoskeleton and spontaneous motion. The first study towards such a synthetic cell model was already performed in 1989 by Cortese et al. They encapsulated actin filaments and actin-binding proteins, such as filamin, into GUVs of 20 μm diameter. K^+^ ions were introduced into the vesicles by ionophores, thus triggering actin polymerisation. This polymerisation process was observed to induce morphological changes of the initially spherical vesicles towards irregular, asymmetric shapes [55]. Sackmann and co-workers later studied GUVs with enclosed actin networks, which were formed by spontaneous swelling in a buffer containing actin monomers. They also observed shape transitions in these model cells following actin polymerisation, but osmotic effects due to the addition of MgCl_2_ were also found to account for these changes [56]. In a subsequent study, self-assembly of thin actin shells beneath the lipid membranes of GUVs was accomplished. Buckling and blister formation of the composite actin–lipid shells were observed, which are typical shape changes in natural cell membranes, too [57]. The binding of actin filaments to positively charged lipid monolayers was further investigated by film balance in combination with neutron reflectivity. Filamentous actin adsorbed well to lipid membranes, whereas no binding was detected for monomeric actin. In dependence of the salt concentration and surface charge density of the lipid monolayer the adsorbed, filamentous actin film had a thickness between 69 and 84 Å [58].

**Figure 4:**
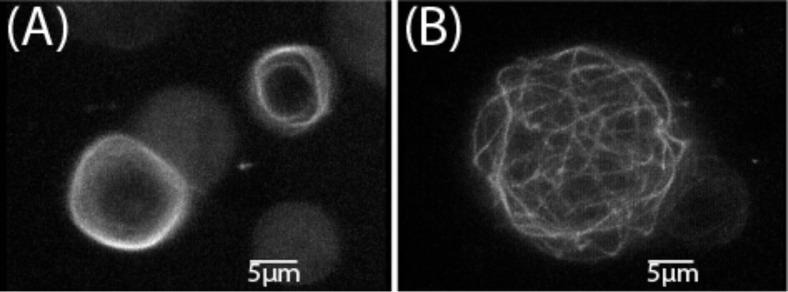
Dependence of actin/α-actinin network structures on the vesicle size. The 3D reconstructions of networks by confocal fluorescence microscopy (at a temperature of 4 °C). (A) Examples of rings obtained in vesicles of diameter *d* < 12 μm. (B) Spiderweblike networks formed in vesicles with diameter *d* > 12 μm (Reprinted with permission from [60]. Copyright (2002) American Physical Society.)

When self-assembled actin networks were encaged in electroformed GUVs, different actin cortex structures were obtained in dependence of the GUV size: for vesicle diameters smaller than the persistence length of actin (≤ 15 μm) a fuzzy cortex formed while larger vesicles yielded a homogeneous network as confirmed by magnetic tweezers [59]. With actin cortices in GUVs, which were cross-linked by α-actinin and filamin, randomly linked networks were observed at 25 °C. For temperatures below 15 °C a transformation into spiderweblike or ringlike networks was observed (Figure 4) [60]. This polymorphism was also found to depend on the vesicle size.

Pautot et al. developed an inverted emulsion technique, which assembles two independently formed lipid monolayers into unilamellar vesicles with a high yield [61]. By using this „droplet transfer“ approach, actin could be efficiently encapsulated in unilamellar vesicles with sizes ranging from 100 nm to 1 μm. Actin polymerisation was induced by the addition of magnesium, and the polymerisation kinetics were unaffected by the encapsulation. Later, inverted emulsion was used to polymerise actin at the inner membrane of larger vesicles with sizes between 1 and 8 μm. This approach preserved the integrity of actin, and polymerisation was triggered by ATP and high salt concentrations [62]. When a continuous actin shell formed at the inner lipid membrane the spreading behaviour of these proteoliposomes on histidine-coated glass slides was reminiscent of a natural cell. The mechanical properties of the actin containing vesicles were found to be mainly governed by the density of the cortical shell [63]. Recently, the droplet transfer technique was extended further to encapsulate filaments of bacterial cytoskeletal proteins, such as MreB and FtsZ, into liposomes [64–65]. Compared to other vesicle preparation techniques, this approach offers a high encapsulation efficiency and good control over protein entrapment without a loss of activity [47].

**Figure 5:**
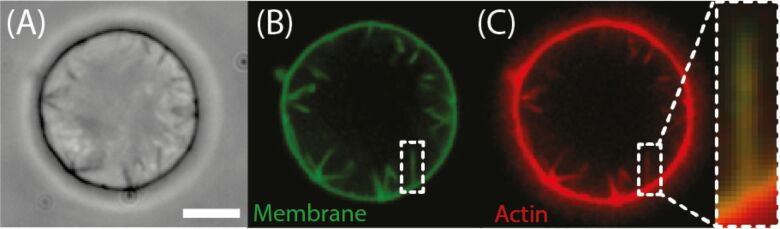
Thin actin protrusions emerge from dendritic actin networks. Phase-contrast (A) and spinning-disc confocal images (B) of lipid membrane (green) and (C) actin (red) show multiple protrusions in the lumen of a GUV. Overlay of the fluorescence images confirms that the membrane protrusions are supported by actin filaments. The scale bar represents 5 μm. (Reprinted with permission from [67]. Copyright (2008) Nature Publishing Group.)

Later, dynamic, branched actin networks were reconstituted on the outside of GUVs by Liu et al. With this model system they demonstrated that actin triggers both temporal and spatial rearrangement of components in the lipid bilayer, thus acting as a membrane domain switch [66]. This study was taken further by assembling dendritic actin networks inside GUVs to study the interaction between actin network growth and deformation of membranes [67]. It was observed that actin-based protrusions formed inside the GUVs, which showed a strong resemblance to cellular filopodia (Figure 5). Liu and co-workers concluded that the lipid membrane also plays an active part in organising actin networks. Already in 1999, Miyata et al. had observed similar protrusive formations at the outside of GUVs with encaged actin filaments. These protrusions developed within 30 to 100 s after KCl was introduced into the GUVs by electroporation and were also evoked by the inner actin polymerisation [68].

Actin filaments have also been encapsulated in giant liposomes together with the molecular motor heavy meromyosin. Without myosin, actin filaments were distributed homogeneously in the liposomes in an unordered manner. In the presence of actin-cross-linking proteins, self-organised actin structures emerged, which were similar to those in living motile cells [69–70]. The liposomes, which incorporated these actin networks, exhibited nonspherical shapes. Experimental protocols on preparing giant liposomes with encapsulated actin, myosin and other cross-linking proteins are discussed in more detail by Takiguchi et al. [69]. In a later study, actomyosin cortices were anchored to the outside or inside of cell-sized liposomes. This arrangement also resulted in shape changes of the biomimetic system. The regulation of morphological changes in such synthetic cells was explained by a balance of actomyosin cortical tension and mechanical resistance to rupture [71].

**Figure 6:**
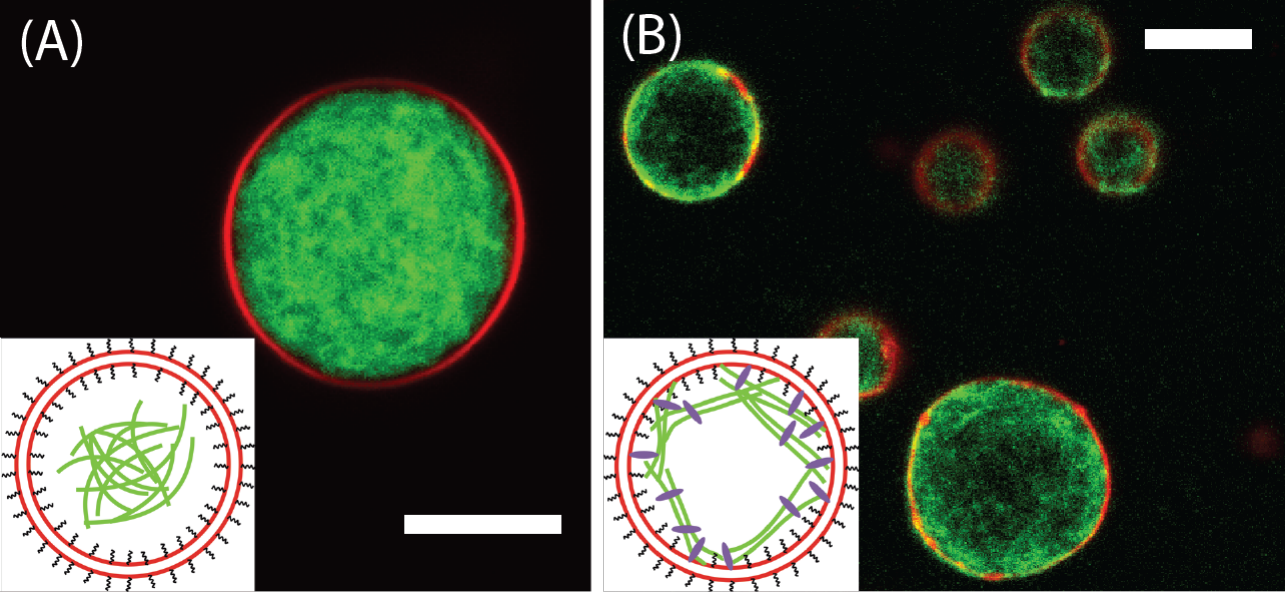
Confocal fluorescence micrographs of giant actin-filled liposomes. Lipid membranes are labelled with rhodamine (red); actin is labelled with AlexaFluor 488 (green). Insets indicate the nature of the actin-membrane interaction. (A) The actin filament solution inside a liposome with an inert membrane (containing PEG-lipids) is homogeneous and displays a depletion zone underneath the membrane. (B) Liposomes, which contain biotinylated lipids, encapsulate networks of biotinylated actin filaments that are coupled to the membrane via biotin–streptavidin bonds. The scale bars represent 10 μm. (Adapted with permission from [72]. Copyright (2011) American Chemical Society.)

For the functional encapsulation of cytoskeletal proteins into lipid vesicles high physiological salt levels are mandatory and the fabrication method should only take a short period of time. These parameters cannot be fulfilled with conventional electroformation, which requires low salt concentrations and takes several hours. Recently, two novel methods were introduced, which overcome these problems of conventional vesicle formation and can incorporate biologically active proteins into GUVs. Gentle hydration of hybrid lipid-agarose films in solutions of cytoskeletal proteins yielded uniform actin and actomyosin networks enclosed in vesicles of 10 to 20 μm diameter (Figure 6) [72]. Actin filaments could also be specifically anchored to the GUV membrane by biotin-streptavidin linkages. This anchorage resulted in the formation of a cortex-like actin structure within the GUVs. However, the GUVs in this study were contaminated by agarose, which adhered to the lipid membrane. A very similar approach for growing GUVs with embedded biomolecules was later presented by Weinberger et al. Lipid precursor films were spread onto polyvinyl alcohol (PVA) films and placed in a swelling buffer containing different biorelevant molecules such as actin. This gel-assisted electroformation of GUVs enabled the fast growth of polymer-free vesicles with high yields. Actin filaments were observed to incorporate into the GUVs, and bundles formed when additional cross-linkers were added to the swelling buffer [73].

### 4. Talin at liposomal membranes

As previously described, talin is a major actin-binding protein, which is a key player in integrin signalling. Moreover, talin anchors the actin microfilament system to the cell membrane and promotes actin polymerisation. With these functions talin can also play a crucial role in the development of synthetic cells from lipid membranes.

Reconstitution of talin into lipid membranes by self-assembly was first achieved by Heise et al. in 1991. Talin was purified from blood platelets and incorporated into vesicles by cyclic freeze-thawing of co-dispersions containing vesicles and talin. This technique yielded a uniform orientation of platelet talin with its large head group pointing to the outer vesicle side [74]. The reconstitution procedure did not change the lipid composition of the vesicles. Charged lipids were found to increase the solubility of talin in the membrane drastically, thus indicating an electrostatic interaction of talin with lipid bilayers. Based on these findings, reconstituted talin in lipid vesicles was later employed to study its interaction with actin filaments. Using fluorescence imaging it could be visualised for the first time that reconstituted talin is able to anchor actin at lipid membranes. Talin was also observed to nucleate actin filaments and to promote growth, as it was reflected by an increase in filament number at the lipid interface [75].

Furthermore, the lipid vesicle model system with reconstituted talin was employed in a study on the function of talin-vinculin complexes. When talin was present, an increase of the actin polymerisation rate was observed, which occurred independently of the presence of vinculin. In calorimetric measurements it was also found that talin, like vinculin, partially inserts into the hydrophobic region of negatively charged lipid bilayers [76]. This finding was later confirmed by light scatter techniques, which yielded a value of 3.3 × 10^5^ M^−1^ for the molar affinity of talin to lipid vesicles [77]. The insertion behaviour of talin into negatively charged lipid bilayers was investigated in more detail by the film balance method combined with fluorescence imaging. With this technique, Sackmann and co-workers showed that fluorescently labelled as well as native talin interacts with negatively charged lipid monolayers [78]. These observations were in good agreement with previous results from differential scanning calorimetry and Fourier transform infrared spectroscopy [74]. Binding assays of talin on lipid membranes later revealed that the protein interacts simultaneously via its 47 kDa polypeptide domain with lipid bilayers and via its 200 kDa domain with actin [79]. Moreover, talin was found to trigger vesicle membrane fusion, which could be monitored using cryoelectron microscopy [80]. Hence, talin is of major importance for understanding cytoskeletal assembly and membrane targeting.

**Figure 7:**
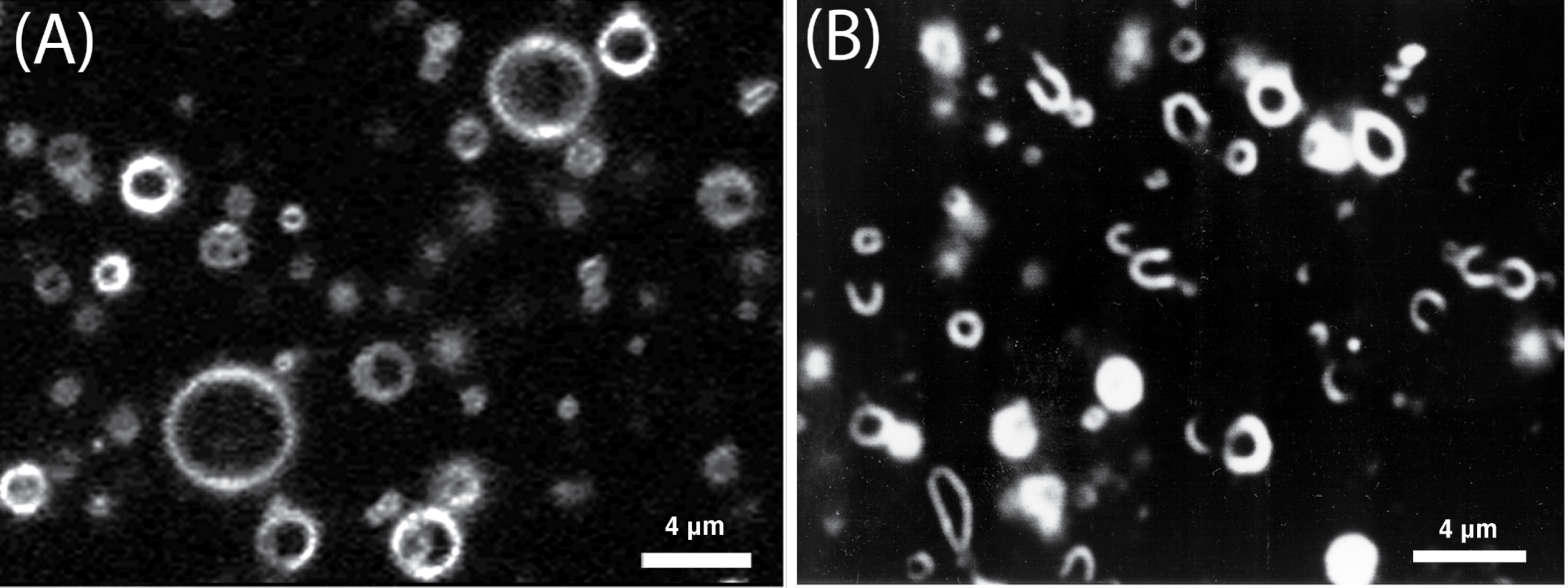
Transformed liposomes observed by dark-field microscopy in the presence of talin. Liposomes used were prepared from phosphatidylethanolamine and phosphatidylglycerol (1:1, mol:mol). (A) Closed spherical liposomes obtained in the absence of talin. (B) Cup-shaped liposomes observed in the presence of 0.4 mM talin. Image courtesy of K. Takiguchi (unpublished).

Further research on the influence of talin on lipid membranes was carried out by Takiguchi and co-workers, who studied the effects of adding talin to liposome solutions. They discovered a membrane-breaking function of talin: Lipid membranes were found to open stable holes and transform into cup-shaped liposomes, which finally turned into lipid bilayers (Figure 7) [81]. Reversion of these morphological changes could be achieved by diluting talin in the surrounding medium, which resulted in the lipid bilayers to transform back into closed liposomes. In future, talin can also be a useful tool for controlled manipulation of liposome morphology, which can play an important role in the development of synthetic cells.

Since the early 2000’s, research on talin reconstituted in lipid bilayers has not been pursued anymore although many fundamental results on cellular functions have previously been obtained from this model system. Nevertheless, small talin head domains have recently been employed to study physiological integrin activation. Ye and co-workers incorporated integrin into liposomes and added talin head domains, which have a size of only 50 kDa. With this reconstituted model system the physiological activation of integrin could be reconstructed in vitro for the first time [82].

## Conclusion

Current research activities on biomimetic model cells with reduced molecular complexity have given fundamental insight into many cellular processes and signalling pathways. In particular incorporating integrins, actin filaments and talin into lipid vesicles has contributed to the current understanding of integrin-mediated cell adhesion and actomyosin-driven motility. Many obstacles in the reconstitution of biologically active membrane proteins into lipid bilayers and the functional encapsulation of actin filaments into vesicles have already been overcome in the past. Yet, there are still many open questions in the field of minimal cellular life, for instance how to incorporate further cytoplasmic proteins, including talin, into giant lipid vesicles. Future research in synthetic cells will certainly pave the way for model systems with tailored properties, which can also play an important role in targeted drug delivery or the development of novel implant materials.
